# Oviposition by mutualistic seed-consuming pollinators reduces fruit abortion in a recently discovered pollination mutualism

**DOI:** 10.1038/srep29886

**Published:** 2016-07-15

**Authors:** Bo Song, Jürg Stöcklin, Yong-Qian Gao, De-Li Peng, Min-Shu Song, Hang Sun

**Affiliations:** 1 Key Laboratory for Plant Diversity and Biogeography of East Asian, Kunming Institute of Botany, Chinese Academy of Sciences, 132 Lanhei Road, Kunming 650201, Yunnan, PR China; 2 Institute of Botany, University of Basel, Schönbeinstr 6, Basel 4056, Switzerland; 3 Yunnan Forestry Technological College, 1 Chuanjin Road, Kunming 650224, Yunnan, PR China

## Abstract

A prerequisite for the evolutionary stability of pollinating seed-consuming mutualisms is that each partner benefits from the association. However, few studies of such mutualism have considered the benefit gained by the pollinators. Here, we determined how the pollinating seed-predators ensure the provisioning of their offspring in the recently discovered mutualism between *Rheum nobile* and *Bradysia* flies. The correlation between flower fate and fly oviposition was examined. Floral traits and patterns of variation in fruit abortion and fly oviposition were investigated to determine whether female flies exhibit preferences for particular flowers when laying eggs. Indole-3-acetic acid (IAA) was quantified to determine whether female flies manipulate host physiology. Flowers that flies oviposited on had a significantly lower probability of fruit abortion compared with intact flowers. Females did not exhibit oviposition preference for any of the floral traits examined. There was no significant correlation between fruit abortion and fly oviposition in terms of either flower position or timing of flowering. IAA concentrations in oviposited flowers were significantly higher than in intact flowers. Our results suggest that oviposition by the mutualistic seed-consuming pollinator *Bradysia* sp., greatly reduces the probability of fruit abortion of its host, *R. nobile*; this may be attributed to the manipulation of host physiology through regulating IAA levels.

The evolutionary stability of mutualisms requires that both partners benefit from the association, otherwise, the interaction will erode into parasitism or predation, or possibly extinction of one or both partners^1^. For decades, evolutionary biologists have searched for the mechanisms that stabilize interspecific mutualisms^2^,^3^,^4^. The mutualistic interaction between pollinating seed-consumers and their host plants, in which pollinators oviposit on flowers and subsequently their larvae consume a portion of the seed crop, are the most iconic systems in the study of interspecific cooperation, in that the success of one species can be expected to affect the success of the other^3^,^5^,^6^. However, empirical studies on the evolutionary stability of pollinating seed-consuming mutualisms are limited to a few species^1^,^3^,^7^,^8^,^9^,^10^. Furthermore, while these studies have paid considerable attention to the positive outcomes for the plants, e.g., how host plants increase their fitness by preventing excessive exploitation by insects, little consideration has been given to the benefit gained by the pollinators.

The ability of females to select suitable oviposition sites is critical to the fitness of most phytophagous insects whose offspring develop and feed at the site where the eggs are laid, since plant resources for the growth and survival of offspring are highly heterogeneous in space and time^11^. For sessile larvae in particular, the progeny either prosper or perish depending on whether the oviposition sites can provide sufficient food resources^12^. Consequently, knowledge about how female adults ensure the provisioning of their offspring at oviposition sites is crucial for understanding the evolutionary stability of pollinating seed-consuming mutualisms^13^.

It has been widely accepted that fruit or seed production is limited by either pollination or resource availability in the absence of flower or seed predation, or adverse environmental conditions^14^. Although pollination is assured by pollinating seed-consuming mutualists, especially with respect to active pollinators, such as *Yucca* moths and *Epicephala* moths^10^,^15^, not all pollinated flowers will subsequently develop into mature fruits (e.g., as a result of fruit abortion), because resource allocation may vary among flowers^16^. Since egg laying by pollinating seed-consumers typically occurs much earlier than seed development, it would be beneficial if ovipositing females could ‘predict’ which flowers were most likely to set seed and have a low probability of fruit abortion, e.g., if they could actively select sites that are most favorable for growth and survival of their offspring. Some ovipositing females have been reported to express preferences for particular floral phenotypes such as flower, or petal size^17^ and corolla tube length^18^ often due to an association of these floral traits with greater rewards. For example, a positive association between larger flowers and a higher likelihood of setting larger fruits and thus providing resources to raise heavier larvae has been reported in the pollinating seed-consuming mutualism *Silene latifolia*/*Hadena bicruris*^19^. Alternatively, specific flower position and flowering phenology have also been reported to be used as cues for oviposition choice by female insects, since reproductive success (e.g., seed production) usually decreases from the proximal (earliest produced) to distal (latest) flowers within inflorescences due to resource limitation^20^,^21^. For example, the pre-dispersal seed predator *Bruchus* beetle preferentially oviposits in fruits with a lower than average probability of abortion and appears to use fruit position and phenology as oviposition cues^12^.

In addition to choosing oviposition sites ‘wisely’, some insect herbivores are thought to be able to manipulate the physiology of host plants by controlling hormones so as to enhance the resources available for larval growth, as seed development is regulated by a variety of hormone signals^22^,^23^,^24^. For example, parasitism of Douglas fir megagametophytes by the seed chalcid *Megastigmus spermotrophus* could prevent parasitized megagametophytes from aborting by inducing similar hormone profiles to normal seed^25^,^26^. The most widely implicated hormone is auxin indole-3-acetic acid (IAA), which can promote cell differentiation and growth, and which has even been suggested to have a direct effect on resource allocation patterns^23^,^27^,^28^,^29^. Some insect herbivores have been found to be able to alter levels of IAA and some even synthesize IAA de novo^24^,^29^,^30^. Furthermore, it has been suggested that the ability of specialist herbivores to manipulate host physiology is stronger than the ability of generalists^31^. Although both oviposition choice and host manipulation to the insect’s benefit have received substantial attention^1^,^23^,^26^,^32^,^33^, the mechanisms by which ovipositing female pollinating seed-consumer ensure the provisioning of their progeny have, to date, been largely ignored.

The interaction between *Rheum nobile* (Polygonaceae) and fly fungus gnat, *Bradysia* sp. (Sciaridae: Diptera) is a recently discovered pollinating seed-consuming mutualism in the high Himalayas^34^. In this system, like other pollinating seed-consuming mutualists reported so far, females of the pollinator, *Bradysia* sp., insert their ovipositor into the style and lay a single egg in each ovary, and pollination is accomplished passively in the process of flower-searching, pollen-feeding and oviposition. The larvae hatch in the developing fruits, each eating the only seed within the parasitized fruit to complete larval development^34^. Although 97% of flowers set fruits, up to 25% of fruits abort, which means that if a female fly lays her egg into a flower that will abort, her offspring will die of starvation. However, our preliminary investigations found that the probability of fruit abortion for flowers in which eggs have been laid by *Bradysia* flies was lower than flowers not selected for oviposition, suggesting that ovipositing flies are good at choosing superior flowers that will ultimately develop into mature fruits, or that they or their offspring are able to manipulate the physiology of their host, *Rheum nobile*, to ensure fruit development so as to provide sufficient food resources. In this study, we determined the role of several floral traits in influencing pollinating seed-predator oviposition choices and the effects of flower position and flowering sequence on fruit set, fruit abortion and oviposition patterns. We also determined the levels of IAA at different developmental stages in both oviposited and intact flowers. We aimed to address the following specific questions: 1) When making oviposition decisions, do *Bradysia* flies discriminate between flowers based on floral traits, position of flowers or flowering phenology? 2) Does oviposition by *Bradysia* flies alter levels of IAA in developing flowers or fruits?

## Results

### Pollination experiments

**Figure 1:**
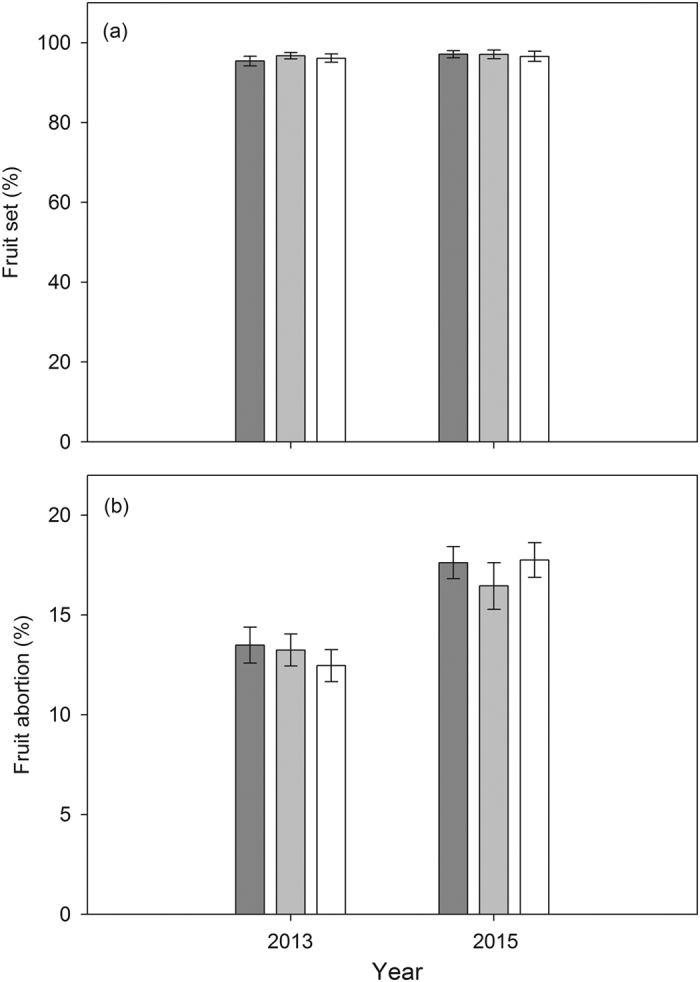
Fruit set (**a**) and fruit abortion rate (**b**) of flowers that were naturally pollinated (*dark gray bars*), hand selfed (*light gray bars*) and hand outcrossed (*white bars*) in 2013 and 2015 in a natural population of *Rheum nobile* at Yongjiongyi, Yunnan in southwest China, 4490 m a.s.l. Data shown are means ± SE (n = 7). Analysis of these data is presented in Table 1.

Flowers subjected to open pollination, manual self-pollination and manual cross-pollination exhibited no difference in fruit set in either year (n.s., Table 1; Fig. 1a), which is consistent with our previous study^34^. Similarly, fruit abortion was not affected by pollination treatment (n.s., Table 1; Fig. 1b). However, the fruit abortion rate differed between the two years (*P* < 0.001, Table 1; Fig. 1b).

### Flower fate and fly oviposition

**Figure 2:**
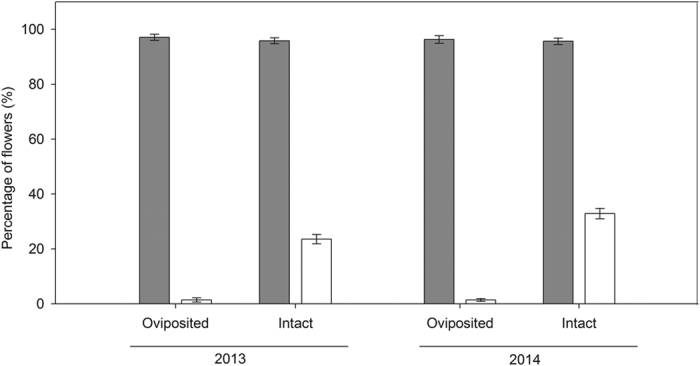
Fruit set (*dark gray bars*) and fruit abortion rate (*white bars*) of flowers that were oviposited by *Bradysia* flies or intact in 2013 and 2014 in a natural population of *Rheum nobile* at Yongjiongyi, Yunnan in southwest China, 4490 m a.s.l. Data shown are means ± SE (n = 7). Analysis of these data is presented in Table 2.

There was no difference in fruit set between oviposited and intact flowers in either year (n.s., Table 2; Fig. 2). Furthermore, no difference was found in stigmatic pollen loads when flies oviposited after feeding on pollen grains compared to flies that only fed on pollen grains (*t* = 0.76, *df* = 38, *P* = 0.45; pollen feeding only: 9.1 ± 1.1 pollen; pollen feeding and ovipositing: 10.3 ± 1.2 pollen), indicating that there was no additional pollination benefit associated with oviposition behavior. However, fruit abortion rate for oviposited flowers was greatly reduced compared to fruit abortion for intact flowers (*P* < 0.001, Table 2; Fig. 2), furthermore, this effect was significantly affected by year (*P* < 0.001; Table 2; Fig. 2). Fruits parasitized by fly larvae were also larger in size than those which were not parasitized for all measured parameters, including length (*F*_1, 118_ = 28.20, *P* <* *0.001), width (*F*_1, 118_ = 127.12, *P* < 0.001) and height of fruit (*F*_1, 118_ = 114.10, *P* < 0.001; Table 3).

### Floral traits and oviposition choices

For all six floral traits measured, there were no differences between flowers that were selected for oviposition by flies and those that were not (corolla length: *F*_1, 198_ = 1.62, *P* = 0.21; corolla width: *F*_1, 198_ = 0.92, *P* = 0.34; ovary length: *F*_1, 198_ = 2.88, *P* = 0.09; ovary width: *F*_1, 198_ = 1.18, *P* = 0.28; stigma lobe length: *F*_1, 198_ = 0.29, *P* = 0.59; stigma lobe width: *F*_1, 198_ = 2.52, *P* = 0.11; Table 3), indicating that *Bradysia* flies have no oviposition preference for any floral traits.

### Positional and temporal variations in fruit set, fruit abortion and oviposition pattern

**Figure 3:**
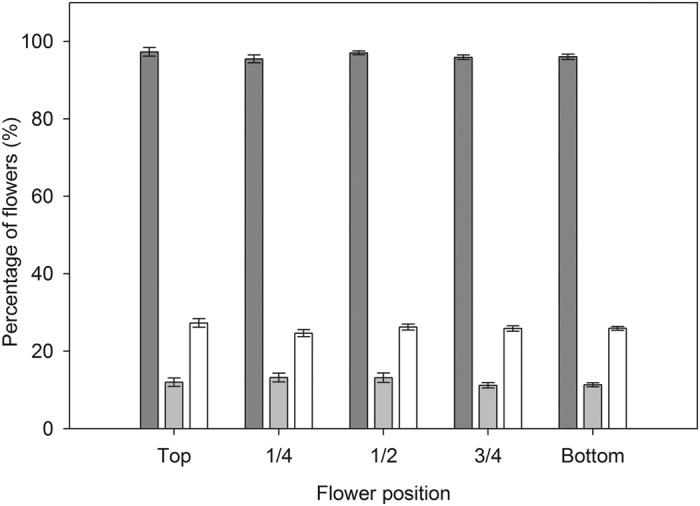
Fruit set (*dark gray bars*), fruit abortion rate (*light gray bars*) and seed predation rate (*white bars*) of flowers across the five sections of the raceme in a natural population of *Rheum nobile* at Yongjiongyi, Yunnan in southwest China, 4490 m a.s.l. Data shown are means ± SE (n = 21).

**Figure 4:**
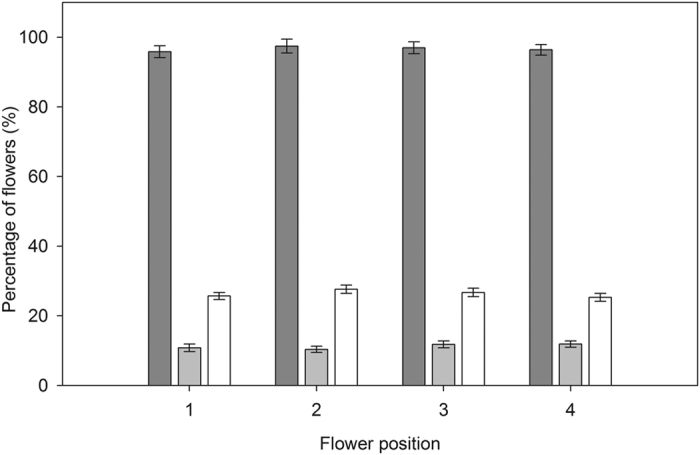
Fruit set (*dark gray bars*), fruit abortion rate (*light gray bars*) and seed predation rate (*white bars*) of flowers across the four sections of the flower head in a natural population of *Rheum nobile* at Yongjiongyi, Yunnan in southwest China, 4490 m a.s.l. Data shown are means ± SE (n = 21).

**Figure 5:**
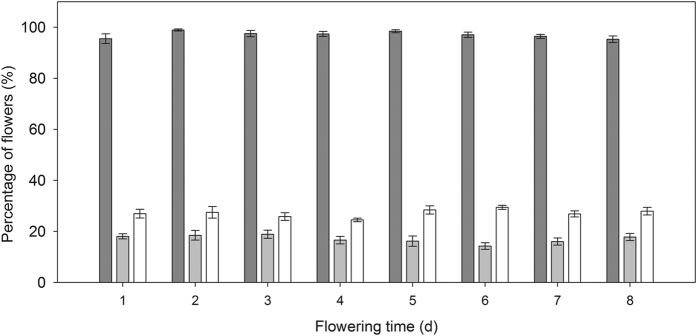
Fruit set (*dark gray bars*), fruit abortion rate (*light gray bars*) and seed predation rate (*white bars*) of flowers that open on different days in a natural population of *Rheum nobile* at Yongjiongyi, Yunnan in southwest China, 4490 m a.s.l. Data shown are means ± SE (n = 8).

There were no significant differences in fruit set and fruit abortion rate across the five sections along the raceme (fruit set: *F*_4, 80_ = 1.99, *P* = 0.10; fruit abortion: *F*_4, 80_* *= 2.27, *P* = 0.07; Fig. 3) nor across the four sections along the flower heads (fruit set: *F*_3, 60_ = 1.12, *P* = 0.31; fruit abortion: *F*_3, 60_ = 1.00, *P* = 0.41; Fig. 4), indicating that a flower’s position did not affect the probability of setting fruit or aborting. Similarly, neither position on the raceme nor position on the flower heads affected the probability of a flower being selected for oviposition (raceme position: *F*_4, 80_ = 1.02, *P* = 0.40; flower head position: *F*_3, 60_ = 1.02, *P* = 0.39; Figs 3 and 4). Correlation analysis showed that there was no significant relationship between fruit abortion rate and oviposition rate along the raceme or flower heads (*r* = 0.06, *P* = 0.56; *r* = 0.03, *P* = 0.81; respectively). In addition, the temporal sequence of flowering had no significant effect on the percentage of flowers that set fruit or that aborted (fruit set: *F*_7, 49_ = 1.61, *P* = 0.16; fruit abortion: *F*_7, 49_ = 1.30, *P* = 0.27; Fig. 5). Similarly, flowers that opened at a different time during flowering of an individual plant had the same chance of being oviposited on (*F*_7, 49_ = 1.35, *P* = 0.25; Fig. 5). No significant correlation was found between fruit abortion rate and oviposition rate in terms of the temporal sequence of flowering (*r* = −0.15, *P* = 0.23).

### Concentration of IAA

**Figure 6:**
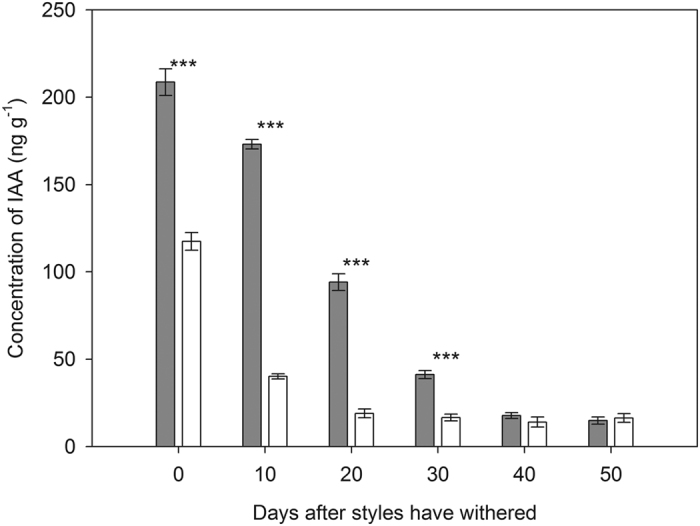
Concentration of indole-3-acetic acid (IAA) in oviposited (*dark gray bars*) and intact (*white bars*) flowers (or fruits) during different developmental stages in a natural population of *Rheum nobile* at Yongjiongyi, Yunnan in southwest China, 4490 m a.s.l. Asterisks denote significant difference at the 0.001 probability level. Data shown are means ± SE (n = 5). Analysis of these data is presented in Table 4.

IAA concentration in oviposited flowers (or fruits) was much higher than that in intact flowers (or fruits) (*P* < 0.001, Table 4; Fig. 6). A significant difference in IAA concentration between oviposited and intact flowers (or fruits) was found until 30 days after the style had withered (Fig. 6). In addition, IAA concentration declined over time during fruit development (50 days), and the decline was disproportionately sharp in intact fruits (*P* < 0.001 for oviposition × day, Table 4; Fig. 6). The difference in IAA concentration between oviposited and intact developing fruits at ten days after the style had withered was 1.5 times higher than immediately after the style had withered.

## Discussion

When larvae cannot move from fruit to fruit on their host plant, their fitness entirely depends on the fate of the flowers on which their mother oviposited^1^,^35^. Therefore, the fate of developing fruits after oviposition may be crucial for the persistence of pollinating seed-consuming mutualisms^11^. Flowers of *Rheum nobile* on which *Bradysia* flies oviposited had a significantly lower probability of fruit abortion than those which female flies did not choose. Previous studies have reported a positive correlation between the oviposition by seed predators on a flower and the flower’s subsequent fate^23^,^26^. The underlying mechanism of this positive effect is still debated. Two putative common mechanisms, oviposition site choice and host manipulation, have been proposed^36^. In this study, we tested the two hypotheses using observational and experimental approaches.

If a plant is resource-limited, it should allocate resources to maturing the highest quality fruits, i.e., those that will produce the largest seeds. In our study, natural pollination occurred in 97% of all flowers, which is similar to manual cross-pollination; furthermore, manual cross-pollination resulted in similar fruit abortion rate as open pollination. These results ruled out the probability that fruit abortion in *R. nobile* is a result of pollen-limitation and suggested that fruit abortion may be due to poor resource allocation^37^. Generally, competition for resources among fruits may be governed by the position and timing of flower initiation on a plant or within an inflorescence^38^,^39^. However, in our study, fruit abortion was random with respect to the position and flowering phenology within inflorescences, i.e., there was no significant positional or temporal variation in fruit abortion. These results suggest that *Bradysia* females cannot use position and flowering phenology as cues to predict which flowers will develop into mature fruits. As a consequence, it is not surprising that there were no positional or temporal variation in oviposition pattern. Two reasons may explain the lack of positional and temporal variation in fruit abortion in this species, similar to what has been demonstrated for a number of other species^40^,^41^,^42^. First, in *R. nobile*, early flowers are distal, late flowers proximal, and thus positional effects of flowers and temporal effects of flowering may offset each other. Secondly, there may be no direct causal relationship between flower position and flowering sequence and fruit abortion. For example, flower position had inconsistent effects on different fitness components in *Tragopogon porrifolius*, with a significant effect on seed mass but not on seed germination^21^. Additional cues may influence oviposition choices by seed predators. It has been suggested that seed predators may choose oviposition sites based on specific floral traits because a positive correlation between floral traits and reproductive success has been demonstrated in a diversity of plant species^17^,^43^. However, although we did not determine the correlation between floral traits and fruit abortion in *R. nobile, Bradysia* flies exhibited no significant oviposition preference for any floral traits examined in the present study, suggesting that female flies did not use specific floral traits as cues for the choice of oviposition sites. Therefore, our results provide no apparent evidence that *Bradysia* flies oviposit selectively in flowers with a low probability of fruit abortion. In fact, in pollinating seed-consuming mutualisms, aside from seed predation costs, the fitness of plants depends on the pollination benefit resulting from the seed predators^5^. It is reasonable to envision that if ovipositing females can predict which flowers will be most likely to develop into mature fruits based on the specific location of flowers or temporal sequence of flowering or specific floral traits, they may preferentially visit flowers with the lowest probability of fruit abortion (i.e., proximal or earliest blooming or large flowers) and ignore other flowers (i.e., distal or latest blooming or small size flowers), causing them not to get pollinated. Rather, plants should complicate developmental patterns and avoid floral traits with reliable oviposition cues, forcing ovipositing females to visit as many flowers as possible and to visit them at random^12^. Although *Bradysia* flies did not oviposit in all flowers, they were equally efficient at pollination of *R. nobile* during flower-searching and pollen-feeding visits and visits that involved oviposition, resulting in stigmatic pollen loads (9–10 pollen grains per stigma) more than sufficient for the fertilization of the single ovule per pistil^34^. Consequently, the lack of consistent positional and temporal variation in fruit abortion and of preference for specific floral traits is beneficial for pollination for *R. nobile*, and contributes to the evolutionary stability of the mutualism. In addition, it is worth noting that there was no significant variation in fruit set with temporal sequences of flowering, suggesting a highly synchrony between flowering phenology and pollinator visits, which further demonstrates the intimate interaction between *R. nobile* and *Bradysia* flies^34^,^44^.

Many insect herbivores have been documented to be able to increase the levels of IAA in host tissues thereby raising the sink status of infested tissues and providing their offspring with food at the plants’ expense^24^,^29^,^45^. In our study, we similarly found that IAA concentration in oviposited flowers (or fruits) was significantly higher than in intact flowers (or fruits). Furthermore, the higher IAA concentration was found particularly in the early stages of fruit development, which corresponds to the time of cell division in the embryo and rapid accumulation of fresh matter in seeds^46^,^47^. The elevated IAA concentration in parasitized fruits of *R. nobile* might enhance localized resource allocation, and thus decrease the risk of fruit abortion^30^,^48^. This was also confirmed by measurements of fruit size: fruits with *Bradysia* larvae were significantly larger than intact fruits. These results suggest that *Bradysia* flies are somehow able to manipulate fruit development of *R. nobile* through regulating IAA concentration. It is known that attack by insect herbivores usually causes a suite of defense responses, induced by various hormones such as jasmonic acid (JA) and abscisic acid (ABA)^27^. IAA has been attributed a role as a negative regulator of JA and ABA^49^. Thus, the elevated IAA concentration in oviposited fruits may help fly offspring to avoid triggering fatal defense responses by their host plant. Further studies exploring the dynamics of such defense-related phytohormones after oviposition by female flies should be conducted to test for such a possibility. In studies aimed at determining the mechanism of ball gall formation on goldenrod, Mapes and Davies^29^ suggested that high levels of IAA in ball galls may result from gall-inducing *Eurosta solidaginis* larvae. In our study, fly larvae did not hatch until the late stage of fruit development^34^, suggesting that the high IAA concentration found in the early stage of fruit development does not result from the seed-consuming larvae themselves and thus can be attributed to the ovipositing females. Further experimental studies are needed to determine whether the elevated IAA concentration in oviposited flowers (or fruits) resulted from prompting IAA production, from changing IAA distribution in the plant through delivery of effectors into the plant cell^24^,^28^,^31^, or from IAA deposition by female flies during oviposition, i.e., IAA synthesized by the flies^50^,^51^.

In conclusion, we have demonstrated that oviposition by the mutualistic seed-consuming pollinator, *Bradysia* sp., can greatly reduce the probability of fruit abortion of its host, *R. nobile*. Our results suggest that female flies ensure the provisioning of their offspring probably by manipulating host physiology through regulating auxin levels rather than by choosing “wisely” flowers that have a low probability of fruit abortion. This is consistent with the hypothesis proposed by Rouault *et al*.^52^ that host manipulation by seed predators is more likely when oviposition occurs before fertilization or seed development because flowers that will have a high or low probability of developing into mature fruits cannot be distinguished from one another at the time of oviposition. To our knowledge, this is the first report of host manipulation in pollinating seed-consuming mutualisms^53^ thereby shedding new and important light on the ecological and evolutionary relationship between plants and their seed-consuming predators. Mutualisms are evolutionarily stable only when each partner can derive benefits from the association^1^. Our results suggest that the seed predators of *R. nobile* are able to assure their benefit. It has been reported that selective fruit abortion plays an important role in reducing seed loss through pre-dispersal seed predation, such as in *Yucca* and *Glochidion*^1^,^3^,^10^. However, our results suggest that *R. nobile* is not able to reduce seed loss due to seed predation through early fruit abortion. This may be due to the single egg oviposited per flower in *R. nobile*^34^, while a variable number of larvae coexist within a single ovary in *Yucca* and *Glochidion*, a necessary requirement for any abortion to be selective^15^,^54^. Further studies determining how plants control seed destruction by the larvae would be particularly useful to understand the evolution and long-term persistence of this recently discovered pollinating seed-consuming mutualism.

## Material and Methods

### Study species and site

*Rheum nobile* is a giant perennial monocarpic herb that grows in the alpine zone (4000–6000 m a. s. l) of the Himalayas and generally inhabits alpine scree^55^. The plants flower from early June to early July and produce a single stout raceme up to 1.5 m in height concealed by large and showy bracts (Fig. S1a). Each raceme is composed of *c*. 45 flower heads bearing 50–400 flowers in clusters along a central axis. Flowering of individual plants lasts for *c*. 10 d and the anthesis of a flower lasts *c*. 3 d. Each flower contains six anthers and one ovule. Stigmas are trifid and receptive 0.5–1 d before anther dehiscence (Fig. S1b). *R. nobile* is self-compatible, but the plant mostly depends on the specialist seed predator fly, *Bradysia* sp., for pollination. After mating on the outer surface of bracts, female flies enter the bracts and look for flowers suitable for pollen consumption and oviposition (Fig. S1c). Each female inserts her ovipositor through the apical pit into the stylar tissue of several flowers and lays one egg in the ovary each time (Fig. S1d). *Bradysia* larvae feed on the mature seeds (Fig. S1e). After completing larval growth, fly larvae exit the fruits and overwinter in the soil as pupae, emerging as adults in June the next year. Larvae have not been observed to exit and reenter other fruits. Fruits mature between late August and late September^34^.

Experiments were undertaken from 2013 to 2015 in a population of *R. nobile* at Yongjiongyi (28°24′ N, 99°55′ E, 4490 m a.s.l.), in Shangri-la Country, Yunnan Province, southwest China. For a full description of the study site, see Song *et al*.^56^.

### Pollination experiments

In order to determine whether fruit set and fruit abortion are affected by pollination and resource availability, seven flowering plants of *R. nobile* were selected randomly in 2013 and in total nine flower heads on each plant were marked. These flower heads were under different bracts and each bract conceals three flower heads. We randomly assigned the three flower heads under each bract to the three pollination treatments described below. Thus, each pollination treatment on each plant included three flower heads that were distributed at different locations on the raceme (basal, middle, and distal). The pollination experiments were as follows:

1. Natural pollination: none of the flowers of a flower head were manipulated.
2. Self-pollination by hand: all flowers of a flower head were enclosed in nylon mesh bags (mesh size = 10 × 10 threads cm^−2^) before anthesis and once these flowers became receptive the flowers were hand-pollinated by rubbing the stigma with fresh dehisced anthers from the same plant. After hand-pollination, the flower heads were bagged again.
3. Cross-pollination by hand: all flowers of a flower head were enclosed in nylon mesh bags (mesh size = 10 × 10 threads cm^−2^) and these flowers were emasculated before anthesis. Receptive flowers were hand-pollinated by rubbing the stigma with fresh dehisced anthers from plants growing *c.* 50 m away and then bagged again.

Mature fruits were collected and fruit set and fruit abortion rate for each flower head were determined in the laboratory. Fruit set was calculated as the number of fertilized flowers/the total number of flowers produced and fruit abortion rate was calculated as the number of immature fruits/the total number of fertilized flowers^56^. For statistical analysis, means across all flower heads of each individual plant and treatment were calculated. This pollination experiment was repeated in the flowering season of 2015.

### Flower fate and fly oviposition

In order to determine the correlation between fruit set, fruit abortion and fly oviposition, seven flowering plants different from the ones used for the pollination experiment were selected randomly in 2013. On each plant, a total of 200 individual flowers from 4–6 flower heads were marked randomly with paper labels before anthesis and were monitored throughout the entire flowering period (from 08:00 to 20:00 h, given that flies are inactive at night)^34^. During the observations, the types of fly visits were recorded individually for all flowers marked (i.e., pollen feeding only vs. pollen feeding and oviposition). When fruits were ripe, all marked flowers were collected and taken to the laboratory to determine fruit set and fruit abortion rate separately for each type of visit on each plant; in the subsequent statistical analysis individual plants were treated as replicates. The experiment was repeated in 2014. In addition, in order to assess the effect of oviposition on pollination efficiency, stigmatic pollen loads following single-pollinator visits where *Bradysia* females either fed on pollen grains only or both fed on pollen grains and oviposited were quantified. Once a flower had been visited by a fly, the type of visit was noted, and the flower was collected and the pollen load on the stigma was counted under a microscope. For each type of visit, 20 flowers from different plants were collected. Since the seeds in fruits with fly larva had been consumed after the fruits were ripe, it is impossible to measure seed mass, so seed mass was evaluated indirectly by measuring fruit size. Six flowering plants were selected randomly in 2015 and these plants were harvested when their fruits were ripe. Ten fruits with and without fly larvae, respectively, on each plant were selected randomly and the fruit size traits were measured, including length, width and height.

### Floral traits and oviposition choices

To determine whether *Bradysia* flies use plant floral traits as cues for oviposition choice, ten flowering plants were selected randomly in 2014 and were monitored throughout the entire flowering period. At the end of the flowering season, for each type of fly visit as described above, ten flowers on each plant were selected randomly for measurement of floral traits. The following floral traits were measured: 1) Length of the corolla; 2) Width of the corolla; 3) Length of the ovary; 4) Width of the ovary; 5) Length of the stigma lobe; and 6) Width of the stigma lobe, given that these traits are frequently associated with pollinator attraction across the angiosperms^57^.

### Positional and temporal variations in fruit set, fruit abortion and oviposition pattern

In order to determine whether flies chose specific locations on the plant for oviposition, and whether those choices were coincident with position effects on fruit set and fruit abortion, fourteen flowering plants were selected randomly in 2013. When the fruits were ripe, these plants were harvested and divided into two groups. In the first group, the raceme was divided into five sections, numbered from top to bottom of the raceme. Section 1 separated the apical flower heads from the rest of the raceme, which was then divided into equal fourths and represented sections 2–5. In each section, three flower heads were selected randomly. In the second group, three flower heads were selected randomly on each plant and these flower heads were divided into four sections using the aforementioned method. In the laboratory, fruit set, fruit abortion rate and seed predation rate were determined separately for each flower head or section. The first group was used to examine the positional variation along the raceme and the second group was used to examine the positional variation within the flower heads. Since seed predation rate did not differ significantly from oviposition rate (*χ*^2^ = 6.0, *df* = 9, *P* = 0.74), we used seed predation rate as a substitute for oviposition rate (as it was for the temporal experiment described below).

In order to determine whether flies chose a specific time of flower opening on the raceme for oviposition, and whether those choices were coincident with temporal effects on fruit set and fruit abortion, eight flowering plants were selected randomly in 2015 and flower development on these plants was monitored throughout the entire flowering period of the plant. On each day, on each plant, we marked *c*. 80 flowers that had just opened: the exact number was dependent on the number of flowers that had opened on a particular day. When fruits were ripe, all marked flowers were collected and taken to the laboratory to determine fruit set, fruit abortion rate and seed predation rate separately for each day of flower opening on each plant, with each individual plant treated as a replicate in the subsequent statistical analysis. Only the data from the first eight days was used since not all plants flowered after the eighth day.

### Concentration of IAA

In order to determine the concentration of IAA in oviposited and intact flowers, five flowering plants were selected randomly in 2015. Prior to pollination, *c.* 600 flower buds were marked randomly on each plant and were enclosed in nylon mesh bags (mesh size = 10 × 10 threads cm^−2^) to exclude insects; flowers were hand-pollinated once the stigma of marked buds became receptive. In addition, *c.* 600 flowers that had been oviposited by *Bradysia* flies on each plant were also marked randomly. From these flowers samples were collected at ten days intervals and immediately frozen in liquid nitrogen until examined further, i.e., exaction of IAA (see below). The first sampling was conducted once the styles of marked flowers had withered at the end of June and the last sampling was in Mid-August. Thus, samplings included six consecutive stages of flower and fruit development. On each sampling date, eighty flowers or fruits (*c.* 50–150 mg of fresh weight) from each plant and for both oviposition treatments (oviposited vs. intact) were collected. Because fly larvae did not hatch until late August or early September, the samples of flowers or fruits oviposited by flies contained eggs but not larvae.

Samples were ground and c. 1 mg of material was used for the IAA extraction. IAA was extracted and detected following the method previously described by Schmelz *et al*.^58^,^59^. Briefly, carboxylic acid was derivatized to methyl ester using trimethylsilyldiazomethane, which was then isolated using vapor phase extraction and analyzed by GC-MS with isobutene chemical ionization using selected-ion monitoring. Prior to processing, 100 ng of [^2^H_5_] IAA (CDN Isotopes, Pointe-Claire, Quebec, Canada) was added to each sample as an internal standard to account for losses during extraction. Samples were also processed without the derivatization agent to verify that the compound recovered was not already present in the plant material, but was derived from the carboxylic acid. The concentration of Me-IAA in extracts was analyzed by GC-MS with electron ionization and their retention times and mass spectra were compared with an authentic compound. Retention time and mass spectra of Me-IAA recovered from flowers (or fruits) oviposited by flies or intact matched those of pure standards, confirming the identity of Me-IAA in our samples.

### Data analysis

In order to test the differences in fruit set and fruit abortion rate between oviposited and intact flowers, and among open pollinated, manually self-pollinated and manually cross-pollinated flowers in the two years, two-way ANOVA was used. Repeated measures ANOVA was used to test the difference in fruit set, fruit abortion rate and oviposition rate across the temporal sequence of flowering, and to test the difference in concentration of IAA between the oviposited and intact flowers (or fruits) during different developmental stages^39^. The effect of position on fruit set, fruit abortion rate and oviposition rate along the raceme or within flower heads was tested using a mixed effect model, with position as a fixed factor and flower head nested within plant as a random factor. A mixed effect model was also used to compare the floral traits and fruit size traits of oviposited and intact flowers, with oviposition as a fixed factor and flower nested within plant as a random factor. Pearson’s correlation test was used to calculate the correlation between fruit abortion rate and oviposition rate. An independent sample *t* test was used to test the differences in number of pollen grains of oviposited and intact flowers, and the difference in IAA concentration between the two oviposition treatments at each collection date. Analyses were performed with SAS statistical software (SAS Institute), with measured variables presented as means ± SE.

## Additional Information

**How to cite this article**: Song, B. *et al*. Oviposition by mutualistic seed-consuming pollinators reduces fruit abortion in a recently discovered pollination mutualism. *Sci. Rep.* **6**, 29886; doi: 10.1038/srep29886 (2016).

## Supplementary Material

Supplementary Information

## Figures and Tables

**Table 1:** Two-way ANOVA of the effects of pollination (open pollination, manual self-pollination and manual cross-pollination) and year on fruit set and fruit abortion rate in a natural population of *Rheum nobile* at Yongjiongyi, Yunnan in southwest China, 4490 m a.s.l.

**Effect**	**Sum of squares**	**df**	***F***	***P***
Fruit set				
Pollination	<0.001	2	0.24	0.80
Year	0.001	1	1.03	0.31
Pollination × year	<0.001	2	0.28	0.76
Error	0.03	36		
Fruit abortion rate				
Pollination	<0.001	2	0.31	0.73
Year	0.02	1	31.84	<0.001
Pollination × year	0.001	2	0.64	0.53
Error	0.20	36		

**Table 2:** Two-way ANOVA of the effects of oviposition (with and without) and year on fruit set and fruit abortion rate in a natural population of *Rheum nobile* at Yongjiongyi, Yunnan in southwest China, 4490 m a.s.l.

**Effect**	**Sum of squares**	**df**	**F**	**P**
Fruit set				
Oviposition	0.001	1	0.68	0.42
Year	0.002	1	0.16	0.69
Oviposition × year	6.4 × 10^−5^	1	0.07	0.80
Error	0.03	24		
Fruit abortion rate				
Oviposition	0.40	1	287.24	<0.001
Year	0.05	1	33.26	<0.001
Oviposition × year	0.04	1	30.23	<0.001
Error	0.03	24		

**Table 3:** Size of floral and fruit traits (means* *±* *SE mm) between flowers that were oviposited by flies or were intact in a natural population of *Rheum nobile* at Yongjiongyi, Yunnan in southwest China, 4490 m a.s.l.

	**Oviposited**	**Intact**
Corolla length	2.64 ± 0.04	2.57 ± 0.03
Corolla width	2.56 ± 0.03	2.52 ± 0.03
Ovary length	1.32 ± 0.01	1.30 ± 0.01
Ovary width	0.91 ± 0.01	0.94 ± 0.02
Stigma lobe length	0.24 ± 0.003	0.23 ± 0.003
Stigma lobe width	0.25 ± 0.003	0.25 ± 0.003
Fruit length	7.25 ± 0.06	6.83 ± 0.05
Fruit width	6.17 ± 0.10	5.07 ± 0.07
Fruit height	5.38 ± 0.08	4.33 ± 0.06

**Table 4:** Repeated measures ANOVA of the effects of oviposition on the concentration of indole-3-acetic (IAA) in different developmental stages of flowers (or fruits) in a natural population of *Rheum nobile* at Yongjiongyi, Yunnan in southwest China, 4490 m a.s.l.

**Effect**	**Sum of squares**	**df**	***F***	***P***
Oviposition	44315.90	1	355.97	<0.001
Day	175720.73	5	681.54	<0.001
Oviposition × Day	36308.56	5	140.82	<0.001
Error	995.96	8		
