# Supplementary material for: Oviposition by mutualistic seed-consuming pollinators reduces fruit abortion in a recently discovered pollination mutualism
Source: Sci Rep. 2016 Jul 15;6:29886. doi: 10.1038/srep29886 (PMC4945934; doi:10.1038/srep29886)
Supplement: Supplementary Information [file srep29886-s1.doc]

**Oviposition by mutualistic seed-consuming pollinators reduces fruit abortion in a recently discovered pollination mutualism**

Bo Song^1^, Jürg Stöcklin^2^, Yong-Qian Gao^3^, De-Li Peng^1^, Min-Shu Song^1^, Hang Sun^1*^

^1^ Key Laboratory for Plant Diversity and Biogeography of East Asian, Kunming Institute of Botany, Chinese Academy of Sciences, 132 Lanhei Road, Kunming 650201, Yunnan, PR China;

^2^ Institute of Botany, University of Basel, Schönbeinstr 6, Basel 4056, Switzerland;

^3^ Yunnan Forestry Technological College, 1 Chuanjin Road, Kunming 650224, Yunnan, PR China

*Correspondence author: hsun@mail.kib.ac.cn; Fax: +86-871-65215002

**Figure legends**

**Fig. S1** *Rheum nobile* and its pollinator. (a) A plant at the anthesis stage. (b) A stigma. (c) A female fly visiting a flower. (d) An ovipositing female fly. (e) A fruit infested by a fly larva (modified from Song *et al*. ^34^).


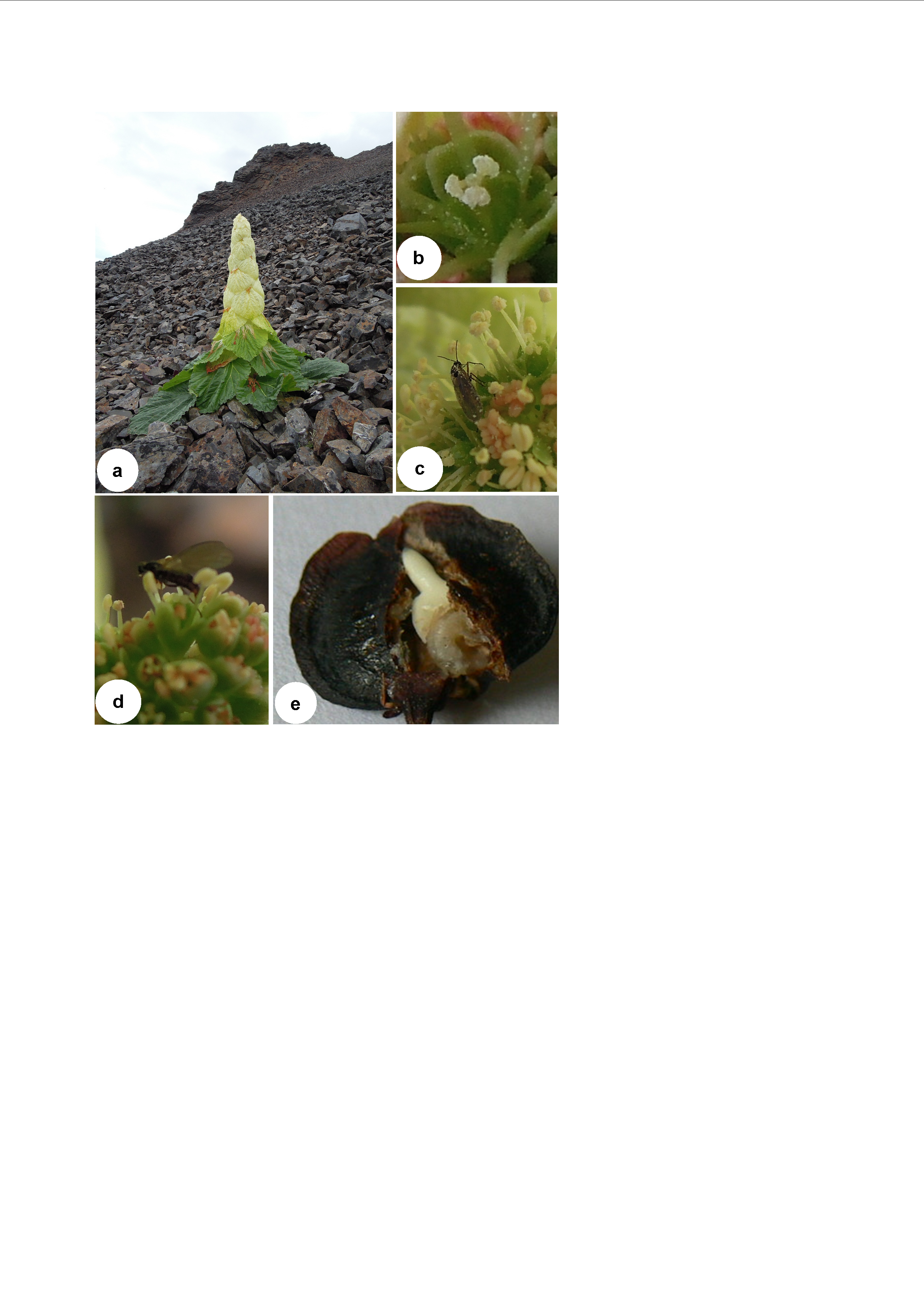


**Figure. S1**
